# Factors associated with prominent vessel sign on susceptibility-weighted imaging in acute ischemic stroke

**DOI:** 10.1038/s41598-021-84269-8

**Published:** 2021-03-11

**Authors:** Hai-fei Jiang, Yi-qun Zhang, Jiang-xia Pang, Pei-ning Shao, Han-cheng Qiu, Ao-fei Liu, Chen Li, Min Jin, Feng-yuan Man, Wei-jian Jiang

**Affiliations:** 1 Medical College of Soochow University, Suzhou, 215123 China; 2 Department of Neurology, Tongzhou People’s Hospital, Nantong, 226300 China; 3 New Era Stroke Care and Research Institute, The PLA Rocket Force Characteristic Medical Center, Beijing, 100088 China

**Keywords:** Medical research, Neurology

## Abstract

The prominent vessel sign (PVS) on susceptibility-weighted imaging (SWI) is not displayed in all cases of acute ischemia. We aimed to investigate the factors associated with the presence of PVS in stroke patients. Consecutive ischemic stroke patients admitted within 24 h from symptom onset underwent emergency multimodal MRI at admission. Associated factors for the presence of PVS were analyzed using univariate analyses and multivariable logistic regression analyses. A total of 218 patients were enrolled. The occurrence rate of PVS was 55.5%. Univariate analyses showed significant differences between PVS-positive group and PVS-negative group in age, history of coronary heart disease, baseline NIHSS scores, total cholesterol, hemoglobin, anterior circulation infarct, large vessel occlusion, and cardioembolism. Multivariable logistic regression analyses revealed that the independent factors associated with PVS were anterior circulation infarct (odds ratio [OR] 13.7; 95% confidence interval [CI] 3.5–53.3), large vessel occlusion (OR 123.3; 95% CI 33.7–451.5), and cardioembolism (OR 5.6; 95% CI 2.1–15.3). Anterior circulation infarct, large vessel occlusion, and cardioembolism are independently associated with the presence of PVS on SWI.

## Introduction

Susceptibility-weighted imaging (SWI) is a magnetic resonance (MR) sequence which exploits the differences of magnetic susceptibility between tissues for imaging^1^. It is a high-resolution, three-dimensional, gradient-echo T2* MR technique that is highly sensitive to both paramagnetic and diamagnetic substances. It has become a useful clinical tool in the field of cerebrovascular diseases. The applications of SWI include the detection of intracerebral haemorrhage, the identification of intra-arterial thrombus, the diagnosis of occult vascular malformation, and the assessment of cerebral haemodynamics following stroke^2^.

The prominent vessel sign (PVS) on SWI refers to asymmetric multiple hypointense vessels in the area of cerebral ischemia^3,4^. It is widely accepted that the PVS is caused by the increased oxygen extraction fraction. In acute ischemic stroke, when blood flow is significantly decreased, the oxygen extraction fraction of the involved brain tissue is elevated, leading to an increase in deoxyhemoglobin in veins and capillaries. Deoxyhemoglobin is a paramagnetic substance with high magnetic susceptibility, which shows the PVS on SWI^5–8^.

Recently, the clinical significance and application of PVS have stirred a lot of interest. Several studies showed that PVS is a sign of clinically relevant hypoperfusion, and it can be used to predict infarct growth and poor outcome^9–13^. It is even thought to be an alternative to perfusion-weighted imaging (PWI)^10^. However, the clinical application of PVS is limited because PVS is not displayed in all patients with acute ischemic stroke. A meta-analysis showed that the presence of PVS ranged from 34 to 100%^14^. The factors related to the presence of PVS are still unclear at present. Therefore, the aim of our study was to investigate the factors associated with PVS in patients with acute ischemic stroke.

## Materials and methods

### Subjects

This was a prospective study conducted in our hospital between August 2013 and August 2017. Consecutive ischemic stroke patients admitted within 24 h from symptom onset underwent emergency multimodal MRI at admission. The inspection sequences consisted of conventional sequences (T1-weighted imaging [T1WI], T2-weighted imaging [T2WI], fluid-attenuated inversion recovery [FLAIR]), diffusion-weighted imaging (DWI), time-of-flight magnetic resonance angiography (TOF-MRA), three-dimensional sampling perfection with application-optimized contrasts by using different flip angle evolution (3D-SPACE), PWI, and SWI. Treatment was not delayed as intravenous thrombolysis was performed in the scanning room using SpaceStation MRI (B. Braun Melsungen AG, Germany), which protects the imaging process against disturbances caused by the infusion pumps.

Patients were included if they met the following inclusion criteria: (1) aged 18 years or older; (2) acute ischemic stroke confirmed by DWI and apparent diffusion coefficient maps; and (3) DWI, TOF-MRA, and SWI were successfully completed within 24 h after symptom onset. The exclusion criteria were as follows: (1) patients who were clinically unstable, required close monitoring, or were moribund; (2) patients with MRI contraindications (heart pacemaker, metallic implant, or severe claustrophobia); (3) patients with any contraindications to intravenous administration of gadolinium (renal failure, pregnancy, or allergy); (4) insufficient image quality due to motion artifact; and (5) patients with no interest in participation. The study protocol was approved by The Ethics Committee of PLA Rocket Force Characteristic Medical Center (approval number: KY2013031) and informed consent was obtained from all participants or their legal guardians. Good Clinical Practice guidelines in accordance with the Declaration of Helsinki were used and the privacy of patients was strictly protected.

### MRI protocol

Patients were imaged on the Siemens Skyra 3.0-T MRI system (Siemens, Germany). The MRI protocol has been reported in our previously published study^15^. DWI was performed with the following parameters: *b*-value 0 and 1000 s/mm^2^; repetition time (TR) 4300 ms; echo time (TE) 98 ms; matrix 173 × 192 pixels; and field of view (FOV) 220 × 220 mm. TOF-MRA was performed using the following parameters: TR 20 ms; TE 3.43 ms; flip angle 18°; matrix 230 × 320 pixels; FOV 192 × 240 mm; slice thickness 1 mm; and 102 slices. For SWI, the magnitude and phase images were obtained with the following parameters: TR 27 ms; TE 20 ms; flip angle 15°; matrix 138 × 256 pixels; FOV 168 × 300 mm; slice thickness 1.5 mm; and 80 slices. Minimum intensity projection images were reconstructed with a thickness of 12 mm.

### Data collection and analysis

A standardized case report form was established for clinical data collection and data management. The following clinical variables were recorded prospectively: demographics (age, gender, and body mass index [BMI]); medical history (hypertension, diabetes, coronary heart disease, congestive heart failure, previous ischemic stroke, and peripheral artery disease); medication history (antiplatelet therapy and statin treatment); life style (cigarette smoking and alcohol consumption); and clinical features (baseline National Institutes of Health Stroke Scale [NIHSS] score, mean arterial pressure, fasting glucose, total cholesterol, triglyceride, hemoglobin, serum creatinine, cardioembolism, and time from onset to MRI).

**Figure 1:**
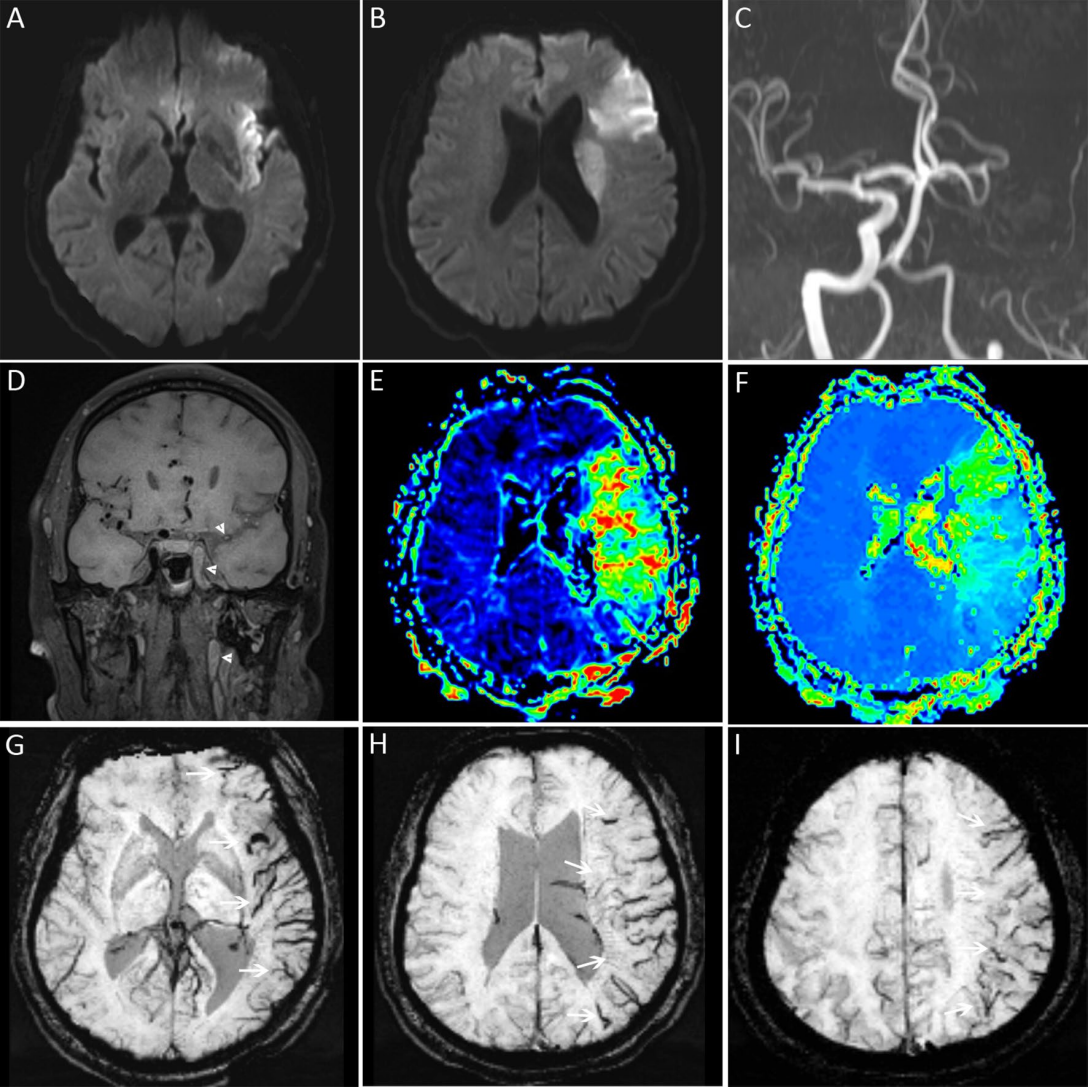
Prominent vessel sign was visible in a patient with anterior circulation stroke due to large vessel occlusion. DWI (**A**,**B**) revealed restricted diffusion in the left insular, frontal, and paraventricular areas. MRA (**C**) and 3D-SPACE (**D**) showed occlusion of the left internal carotid and middle cerebral arteries (white arrowheads). The MTT (**E**) and TTP (**F**) maps indicated hypoperfusion in the left middle cerebral artery territory. Asymmetric prominent hypointense vessels (white arrows), known as prominent vessel sign, appeared on SWI (**G**–**I**) in the affected regions.

All images generated were saved on compact disk-read only memory together with the visualization software (Syngo FastView, Siemens, Germany). This software is freely accessible at https://www.siemens-healthineers.com/medical-imaging-it/advanced-visualization-solutions/syngo-fastview. Two readers (one with 9 years and another with 12 years of experience in stroke imaging) independently reviewed all MRI images without access to clinical data, and disagreements were resolved by discussion. PVS was defined as a local prominence of hypointense vessels on SWI, with either increased vessel number or diameter in the target area, relative to the non-target area (Fig. 1). All patients were divided into PVS-positive group and PVS-negative group based on the presence or absence of the prominent hypointense vessels. Unilateral lesion and anterior circulation infarct were recognized by DWI and apparent diffusion coefficient maps. Large vessel occlusion was identified on TOF-MRA and defined as occlusion of large arteries (internal carotid artery, M1 or M2 segment of middle cerebral artery, A1 segment of anterior cerebral artery, vertebral artery, basilar artery, and P1 segment of posterior cerebral artery).

### Statistical analyses

Statistical analyses were performed with SAS software (version 9.2; SAS Institute, Cary, NC, USA). *P* < 0.05 was considered statistically significant.

The distribution of the data was analyzed using the Kolmogorov–Smirnov test. Continuous variables were described as mean ± standard deviation (SD) or median with interquartile range (IQR) according to the sample distribution. Categorical variables were expressed as number and percentage. The agreement between the two readers was determined with Cohen’s κ. For univariate analyses, independent two-sample *t*-test or Mann–Whitney *U* test was used for continuous variables, and chi-square test was used for categorical variables. Variables with a *P* < 0.10 in univariate analyses were introduced into the multivariable logistic regression model in a stepwise manner.

## Results

A total of 218 consecutive patients were enrolled. The baseline characteristics are summarized in Table 1. The patients consisted of 129 (59.2%) men and 89 (40.8%) women. The mean age was 66 ± 14 years. The median baseline NIHSS score was 6 (IQR 3–11). 76 (34.9%) patients were identified as cardioembolism. The median onset-to-MRI time was 3.4 h (IQR 2.6–4.5 h).

**Table 1:** Univariate analysis to identify factors associated with prominent vessel sign.

Variable	Total (n = 218)	PVS negative (n = 97)	PVS positive (n = 121)	*P* value
**Demographics**				
Age, years, median (IQR)	67 (57–78)	64 (55–74)	71 (58–79)	0.014
Female, n (%)	89 (40.8)	36 (37.1)	53 (43.8)	0.318
Body mass index, kg/m^2^, median (IQR)	24.5 (22.1–27.2)	24.5 (22.9–26.1)	24.7 (21.5–27.3)	0.984
**Medical history**				
Hypertension, n (%)	147 (67.4)	68 (70.1)	79 (65.3)	0.451
Diabetes, n (%)	55 (25.2)	26 (26.8)	29 (24.0)	0.632
Coronary heart disease, n (%)	35 (16.1)	10 (10.3)	25 (20.7)	0.039
Congestive heart failure, n (%)	6 (2.8)	2 (2.1)	4 (3.3)	0.888
Previous ischemic stroke, n (%)	55 (25.2)	29 (29.9)	26 (21.5)	0.155
Peripheral artery disease, n (%)	6 (2.8)	2 (2.1)	4 (3.3)	0.888
**Medication history**				
Antiplatelet therapy, n (%)	43 (19.7)	18 (18.6)	25 (20.7)	0.698
Stain treatment, n (%)	34 (15.6)	19 (19.6)	15 (12.4)	0.146
**Life style**				
Smoking, n (%)	63 (28.9)	29 (29.9)	34 (28.1)	0.771
Drinking, n (%)	43 (19.7)	24 (24.7)	19 (15.7)	0.096
**Clinical information**				
Baseline NIHSS, median (IQR)	6 (3–11)	4 (2–8)	9 (4–15)	< 0.001
Mean arterial pressure, mmHg, mean ± SD	110 ± 15	110 ± 15	109 ± 16	0.640
Fasting glucose, mmol/L, median (IQR)	7.0 (6.0–8.9)	7.0 (6.0–8.8)	7.0 (6.0–9.0)	0.673
Total cholesterol, mmol/L, median (IQR)	4.2 (3.2–4.9)	4.4 (3.3–5.1)	3.9 (3.0–4.8)	0.014
Triglyceride, mmol/L, median (IQR)	1.5 (1.0–2.8)	1.8 (1.2–2.8)	1.4 (1.0–2.7)	0.066
Hemoglobin, g/L, median (IQR)	139 (128–151)	144 (134–156)	136 (125–146)	0.001
Serum creatinine, μmol/L, median (IQR)	74.0 (62.5–86.9)	72.0 (59.2–84.7)	76.2 (63.3–88.2)	0.149
Time from onset to MRI, min, median (IQR)	203 (154–272)	214 (160–279)	197 (151–267)	0.420
Cardioembolism	76 (34.9)	16 (16.5)	60 (49.6)	< 0.001
**Image features**				
Unilateral lesion, n (%)	183 (83.9)	82 (84.5)	101 (83.5)	0.831
Anterior circulation infarct, n (%)	159 (72.9)	51 (52.6)	108 (89.3)	< 0.001
Large vessel occlusion, n (%)	107 (49.1)	7 (7.2)	100 (82.6)	< 0.001

*IQR* interquartile range; *NIHSS* National Institutes of Health Stroke Scale; *PVS* prominent vessel sign; *SD* standard deviation.

PVS was present in 121 patients. The occurrence rate of PVS was 55.5% (121/218) for all patients, while it was up to 100% (90/90) for anterior circulation stroke patients with large vessel occlusion. Excellent agreement for the detection of PVS on SWI was observed, with a κ value of 0.83 (95% confidence interval [CI] 0.76–0.91).

Univariate analyses showed that patients with PVS had older age and higher NIHSS scores. Total cholesterol and hemoglobin were lower in the PVS-positive group than PVS-negative group. Patients with coronary heart disease, anterior circulation infarct, large vessel occlusion, and cardioembolism were more likely to have PVS.

**Figure 2:**
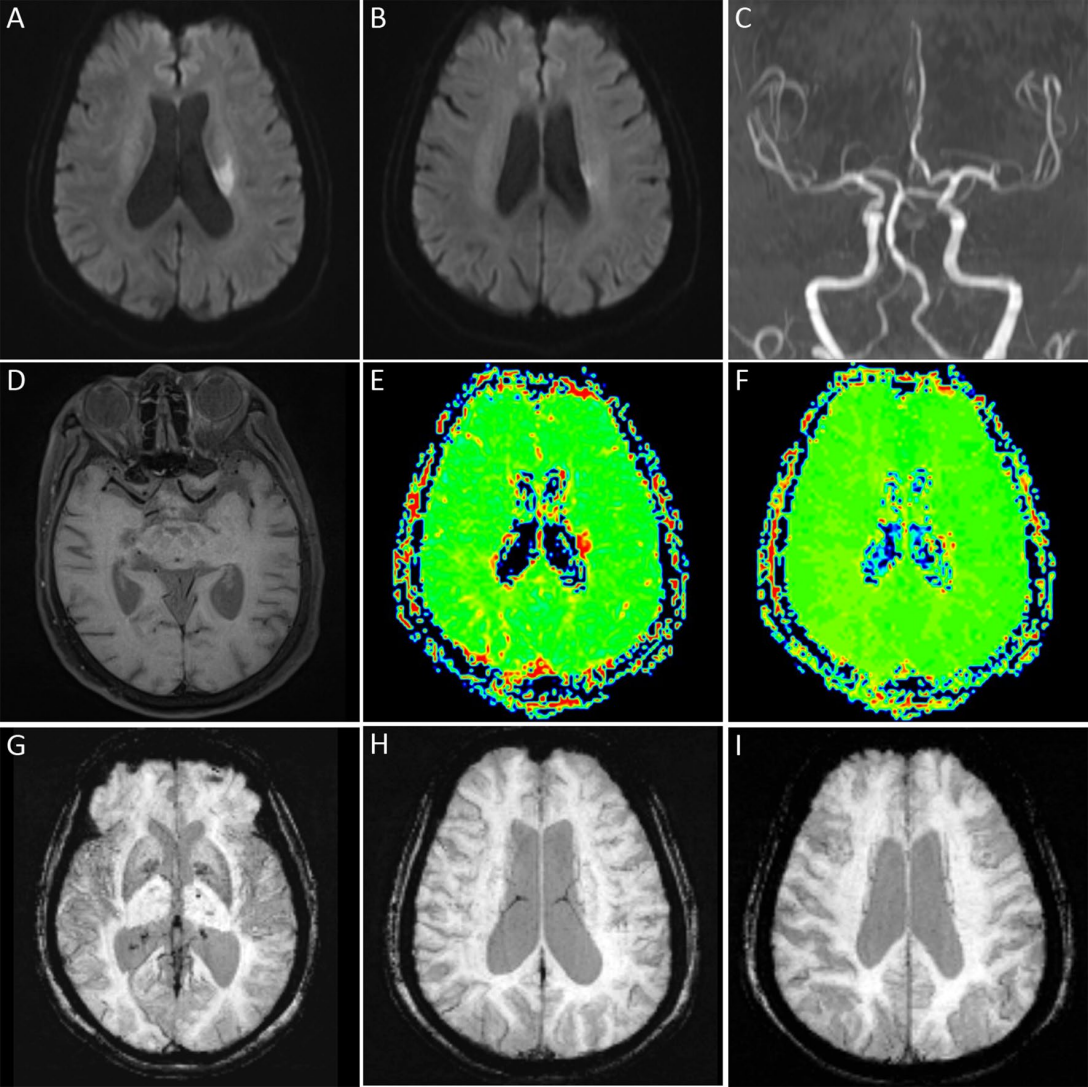
Prominent vessel sign was not displayed in a patient with perforator occlusion in the anterior circulation. DWI (**A**,**B**) showed lacunar infarction in the left paraventricular region. Large vessel occlusion was not seen on MRA (**C**) and 3D-SPACE (**D**). The MTT (**E**) and TTP (**F**) maps indicated a small area of hypoperfusion near the left lateral ventricle. There was no prominent vessel sign on SWI (**G**–**I**).

**Figure 3:**
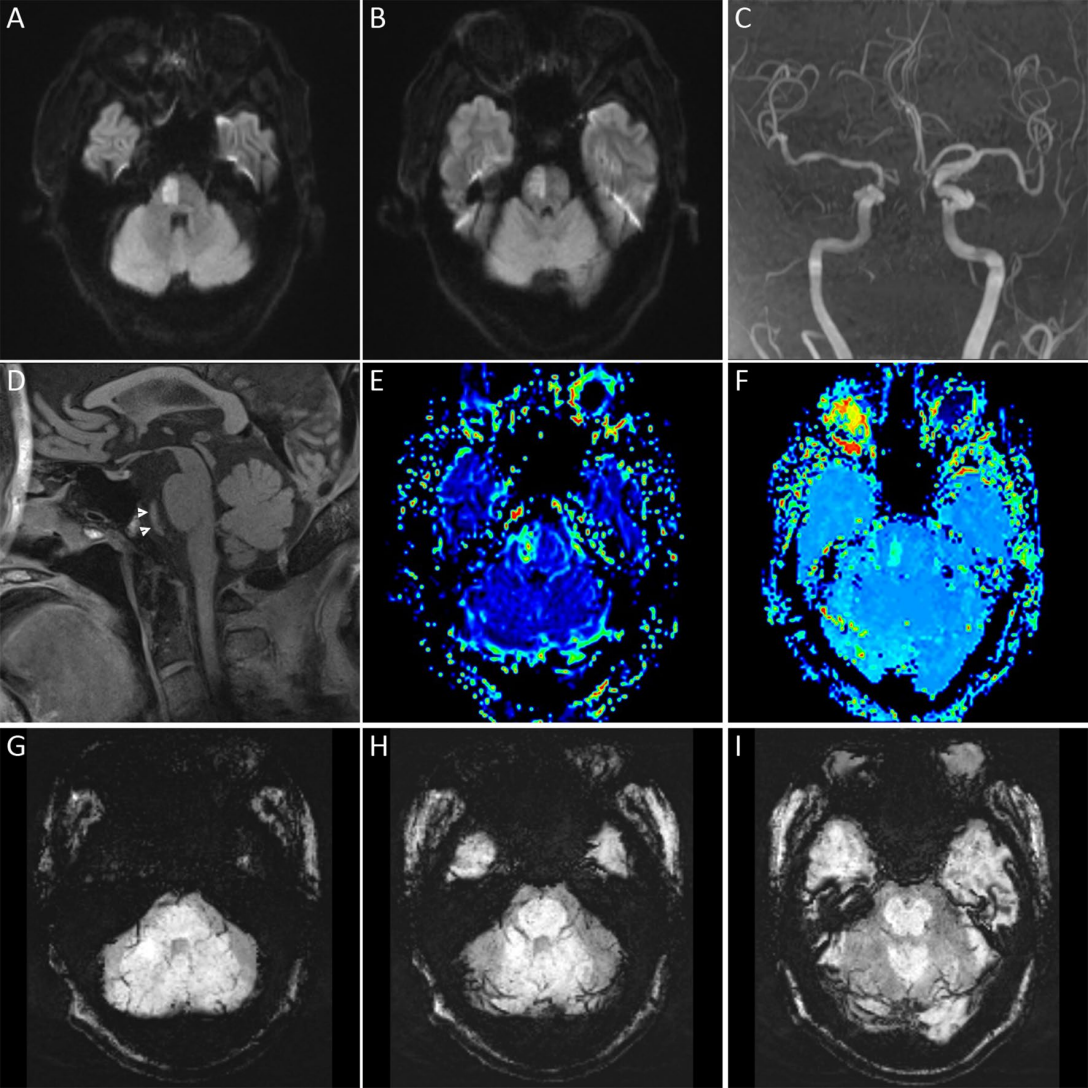
Prominent vessel sign was not displayed in a patient with posterior circulation occlusion. DWI (**A**,**B**) showed acute pontine infarction. MRA (**C**) and 3D-SPACE (**D**) indicated occlusion of the basilar artery (white arrowheads). The MTT (**E**) and TTP (**F**) maps showed hypoperfusion at the infarct site. There was no asymmetric prominent hypointense vessels on SWI (**G**–**I**) in the posterior circulation territory.

On multivariable logistic regression analysis, only cardioembolism (odds ratio [OR], 5.6; 95% CI 2.1–15.3; *P* = 0.001), anterior circulation infarct (OR 13.7; 95% CI 3.5–53.3; *P* < 0.001), and large vessel occlusion (OR 123.3; 95% CI 33.7–451.5; *P* < 0.001) were found to be independently associated with PVS (Table 2, Figs. 2 and 3).

**Table 2:** Independent factors associated with prominent vessel sign.

Variable	OR	95% CI	*P* value
Anterior circulation infarct	13.7	3.5–53.3	< 0.001
Large vessel occlusion	123.3	33.7–451.5	< 0.001
Cardioembolism	5.6	2.1–15.3	0.001

*CI* confidence interval; *OR* odds radio.

## Discussion

A number of terms have been used to describe the finding of asymmetric hypointense vessels on SWI in patients with acute ischemic stroke: prominent hypointense vessel sign, cortical vessel sign, brush sign, deep medullary veins, ipsilateral prominent thalamostriate vein, prominent veins, asymmetrical cortical vein sign, asymmetrical medullary vein sign, asymmetrically prominent cortical veins, prominent cortical veins, and multiple hypointense vessels^11–13,16–20^. Some of these terms are synonymous, and some describe different vessels involved. Since the prominent hypointense vessels may be cortical veins, medullary veins, subependymal veins, or even small arteries containing deoxyhemoglobin, we adopted the term PVS in our study^15^.

The presence of PVS on SWI in patients with acute cerebral infarct is due to the decreased oxygen supply in the area of the responsible artery. In order to obtain more oxygen, ischemic and hypoxic brain tissue compensates by increasing oxygen extraction fraction, resulting in an increase in the proportion of deoxyhemoglobin in local vessels. However, not all patients with acute ischemic stroke have PVS on SWI. At present, some studies have compared the differences of related factors between the PVS-positive group and the PVS-negative group^13,18^. But these were retrospective studies with insufficient sample size and only univariate analyses were used. In this prospective study, all patients completed multimodal MRI within 24 h of symptom onset, with the vast majority (86%) completed within 6 h. Sufficient cases were enrolled and multivariable analyses were used to adjust the influence of confounding factors. Univariate analyses showed that there were significant differences between PVS-positive group and PVS-negative group in age, history of coronary heart disease, baseline NIHSS scores, total cholesterol, hemoglobin, anterior circulation infarct, large vessel occlusion, and cardioembolism. We further conducted multivariable logistic regression analyses, and found that only anterior circulation infarct, large vessel occlusion, and cardioembolism were independently correlated with the presence of PVS.

In our study, the occurrence rate of PVS was 67.9% in patients with anterior circulation infarct. Most previous studies on PVS have focused on anterior circulation stroke. Chen et al.^3^ performed a study which included 22 patients with acute brain infarct in the territory of the middle cerebral artery. PVS was detected in 15 patients. The occurrence rate (68.2%) is almost identical to ours. Our study found that only 22% of patients with posterior circulation infarc had PVS. A retrospective study analyzed 22 MRI of patients with basilar artery occlusion^21^. The authors acknowledged that hypoperfused areas in the anterior circulation can be easily identified by the presence of hypointense veins, while identification of hypoperfused areas in the posterior circulation is more challenging. Because first, the prominent vessels with a large diameter could also appear in regions that are not affected by ischemia, especially in the cerebellum; and second, cortical vessels surrounding the brainstem or the thalamus are often hard to identify due to the small caliber of blood vessels in these regions. In addition, occlusion of some arteries in the posterior circulation may lead to bilateral infarct, making it difficult to identify PVS through asymmetry.

Large vessel occlusion is another factor independently associated with the presence of PVS. Morita et al.^22^ reported that neither cortical vessel sign nor brush sign was found in patients with minor vessel disease. In our study, we found no PVS in patients with lacunar infarct caused by perforator artery occlusion, while the occurrence rate of PVS for anterior circulation stroke patients with large vessel occlusion was 100%. In Morita et al.^22^ study, all 24 (100%) patients with large vessel occlusion (internal carotid artery, M1 or M2 segment) manifested the cortical vessel sign. In Verma et al.^19^ study, of 33 patients with M1 segment occlusion, 32 (97.0%) showed prominent cortical veins. In Liang et al.^4^ study, of 37 anterior circulation infarct patients with large artery occlusion or stenosis, 35 (94.6%) had PVS on SWI. These results are close to ours.

One-third of the patients included in this study had cardioembolic stroke. Among all patients with cardioembolism, 78.9% showed PVS on SWI. We believe that the size of detached thrombus is related to the presence of PVS. When a large embolus causes an intracranial or extracranial occlusion of a large vessel, it can lead to a wide range of cerebral ischemia. When a medium-sized artery in the cerebral cortex, such as the M3 segment of the middle cerebral artery, is suddenly blocked, a moderate range of cortex ischemia can occur. PVS may appear on SWI under both circumstances. However, when a small embolus is detached and embolized in an arteriole, PVS is not shown.

Our study has several limitations. First, it is difficult to detect all unmeasured confounding factors that could explain the appearance of PVS, which it is an inherent issue of observational studies. Two previous studies have shown that collateral circulation is associated with PVS. One study pointed out that good leptomeningeal collateralization correlates with less prominent cortical veins^19^. Another study found the opposite, suggesting that better collateral flow is associated with more extensive hypointense vessels^20^. The relationship between collateral circulation and PVS needs to be further confirmed. Second, PVS was obtained by observation and comparison rather than by objective measurement. Since it is a subjective parameter, there may be bias in the interpretation of images. Quantitative susceptibility mapping is a development of SWI that can visualize veins and quantify blood oxygen saturation by measuring susceptibility values^23–25^. This technique may accurately identify hypointense vessels and provide more quantitative information about cerebral ischemia.

In conclusion, anterior circulation infarct, large vessel occlusion, and cardioembolism are independently associated with the presence of PVS. On SWI, PVS is almost always visible in patients with anterior circulation stroke due to large vessel occlusion.
